# Epidemiology of Glucose-6-Phosphate Dehydrogenase Deficiency in Arab Countries: Insights from a Systematic Review

**DOI:** 10.3390/jcm12206648

**Published:** 2023-10-20

**Authors:** Abdulaziz S. Alangari, Ashraf A. El-Metwally, Abdullah Alanazi, Badr F. Al Khateeb, Hanan M. Al Kadri, Ibtehaj F. Alshdoukhi, Aljohrah I. Aldubikhi, Muzun Alruwaili, Awad Alshahrani

**Affiliations:** 1 College of Public Health and Health Informatics, King Saud bin Abdulaziz University for Health Sciences, Riyadh 11426, Saudi Arabia; 2 King Abdullah International Medical Research Center, Riyadh 11481, Saudi Arabia; 3 Department of Family Medicine, King Abdulaziz Medical City, Ministry of the National Guard-Health Affairs, Riyadh 11426, Saudi Arabia; 4 Department of Obstetrics and Gynecology, King Abdulaziz Medical City, Ministry of the National Guard-Health Affairs, Riyadh 11426, Saudi Arabia; 5 Department of Basic Sciences, College of Science and Health Professions, King Saud bin Abdulaziz University for Health Sciences, Riyadh 14611, Saudi Arabia; 6 College of Health Sciences, Saudi Electronic University, Riyadh 11673, Saudi Arabia; 7 College of Medical Sciences, North Border University, Arar 91431, Saudi Arabia; 8 Department of Medicine, King Abdulaziz Medical City, Ministry of the National Guard-Health Affairs, Riyadh 11426, Saudi Arabia; 9 College of Medicine, King Saud bin Abdulaziz University for Health Sciences, Riyadh 11481, Saudi Arabia

**Keywords:** epidemiology, G6PD deficiency, Arab countries, systematic review, metabolic disorder

## Abstract

Glucose-6-phosphate dehydrogenase (G6PD) deficiency is a common metabolic disorder affecting more than 400 million individuals worldwide. Being an X-linked disorder, the disease is more common among males than females. Various Arab countries estimated the prevalence of G6PD deficiency; however, findings from different countries have not been synthesized collectively. Hence, a systematic review was undertaken to synthesize the findings on the epidemiology of G6PD deficiency in all Arab countries. We performed an electronic systematic literature search based on the eligibility criteria using databases, including MEDLINE, Embase, and CINHAL. The studies included in the review were primary and original research studies assessing the prevalence or incidence, risk factors, or determinants of G6PD deficiency, and published in the English language in a peer-reviewed scientific journal between 2000 and 2022. The systematic review was carried out with the help of an updated PRISMA (Preferred Reporting Items for Systematic Reviews and Meta-Analyses) checklist. After the screening, 23 full texts were finalized for data extraction. The prevalence of G6PD deficiency ranged from 2 to 31% with a greater burden among high-risk populations like neonates with sickle cell anemia. The determinants included males, family history, consanguineous marriages, and geographic regions, which were all risk factors, except for body weight, which was a protective factor. The prevalence of G6PD deficiency varies across Arab countries, with a higher prevalence in males than females. Different regions of Arab countries need to revisit their screening and diagnostic guidelines to detect G6PD deficiency promptly and prevent unnecessary morbidity and mortality among their communities.

## 1. Introduction

Glucose-6-phosphate dehydrogenase (G6PD) deficiency is the most common enzyme deficiency in the world. G6PD deficiency is a genetic disorder, and the gene responsible for encoding G6PD is found on the X-chromosome long arm [1]. Glucose-6-phosphate dehydrogenase is important for the stability of red blood cells [2]. The enzyme catalyzes the first step in the hexose monophosphate pathway of the metabolism of glucose, and generates reduced nicotinamide adenine dinucleotide phosphate, which is necessary to maintain reduced glutathione. The reduced glutathione is important for protecting red blood cells from oxidative stress and fragility [2,3]. While the affected individuals typically remain asymptomatic, factors such as specific foods, antibiotics, and infectious agents causing excessive oxidative stress in the body can elicit a hemolytic crisis (favism), which may either be self-limiting or increase the need for a blood transfusion [4]. G6PD deficiency is one of the major causes of neonatal hyperbilirubinemia, which can eventually lead to kernicterus [5].

G6PD deficiency is a widespread metabolic disorder of red blood cells, and has been found to affect more than 400 million individuals around the world [1,6]. Being an X-linked disorder, the number of deficient males determines the burden of G6PD deficiency in a given population [1]. Since the discovery of G6PD deficiency in 1956, the prevalence and burden of G6PD deficiency have been estimated across the regions of the world [7]. The estimated prevalence is made possible by the accessibility to cheap and rapid screening tests. The disease was first reported in India; however, it is found in Mediterranean, African, and Asian countries, with a prevalence of about 25% in some countries [8].

In populations with high prevalence rates, early detection of enzyme deficiency by neonatal screening is required to take appropriate measures [9,10,11]. Based on the screening and related prevalence, a few countries have established screening programs to prevent severe jaundice in children [9,11]. However, the epidemiology—including prevalence or incidence of the disease, distribution by gender, and risk factors of the disease—may be helpful in developing country-specific screening and diagnostic guidelines. Various countries in the Arab world have attempted to estimate the prevalence of G6PD deficiency. However, the findings from different countries have not been synthesized collectively. Hence, the aim of this systematic review is to review and synthesize findings on the epidemiology of G6PD deficiency in all Arab countries.

## 2. Material and Methods

A systematic review was undertaken to synthesize evidence from published research studies on the epidemiology of glucose-6-phosphate dehydrogenase (G6PD) deficiency in 22 Arab countries. The systematic review was carried out in accordance with the PRISMA (Preferred Reporting Items for Systematic Reviews and Meta-Analyses) statement [12].

### 2.1. Eligibility Criteria

A research study was included in the review if it was a primary and original research study assessing the prevalence or incidence, risk factors, or determinants of G6PD deficiency, and if it was published in the English language in a peer-reviewed scientific journal from 2000 to 2022.

### 2.2. Information Sources and Search Strategy

After employing the above-mentioned eligibility criteria, an electronic systematic literature search was performed using electronic databases, including MEDLINE, Embase, and CINHAL. These databases were searched using a predefined search strategy, having key search terms and combinations of relevant search terms. In addition to searching the databases, the references of the eligible primary research articles were also explored to find additional relevant research studies.

The research articles were scrutinized using a sequence of search terms, and their combinations were chosen based on the proposed research question. Four major concepts were defined, including *Epidemiology*, *Glucose 6 phosphate dehydrogenase deficiency*, and *Arab countries* by specifying the names of the countries. Later, key concepts were combined using Boolean Operators (AND, OR), and truncation (*) was employed to find additional research studies with identical root words. Moreover, indexed keywords in the medical subject headings (MeSH) were employed to guarantee consistent search terms. Below is an example of the search strategy used to identify relevant research articles in the databases:

Example: (Epidemiology*) AND (Glucose 6 phosphate dehydrogenase deficiency or G6PD deficiency) AND (Arab countries) OR (Algeria, Bahrain, the Comoros Islands, Djibouti, Egypt, Iraq, Jordan, Kuwait, Lebanon, Libya, Morocco, Mauritania, Oman, Palestine, Qatar, Saudi Arabia, Somalia, Sudan, Syria, Tunisia, the United Arab Emirates, and Yemen).

### 2.3. Study Selection

**Figure 1:**
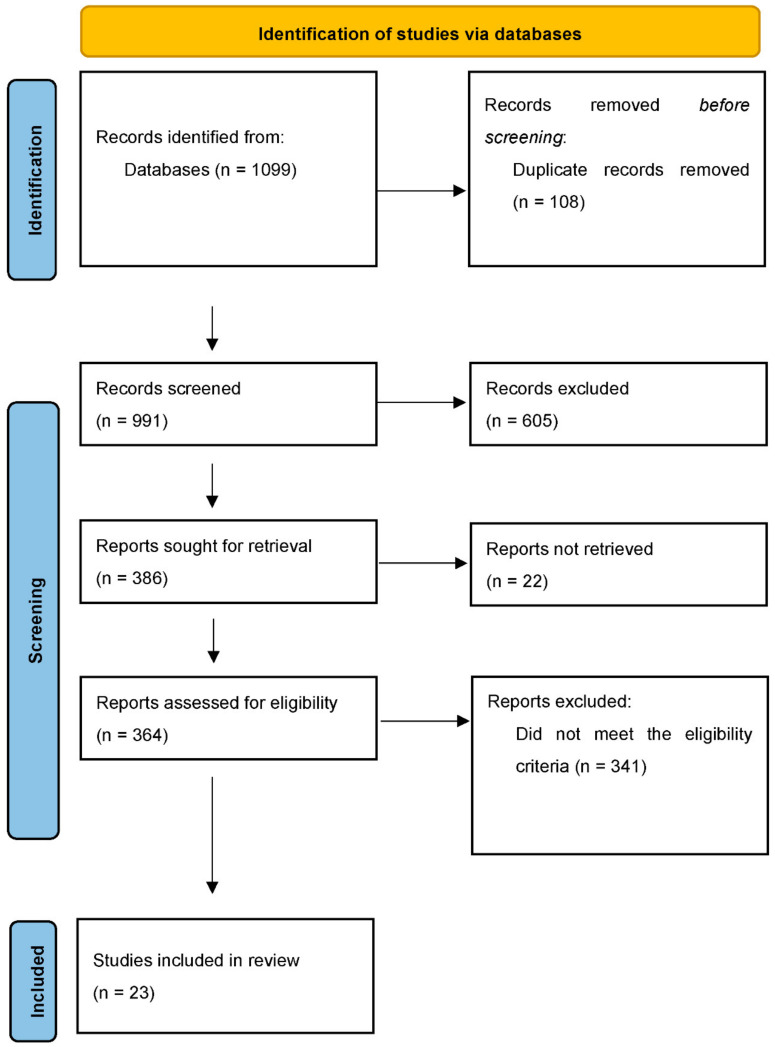
Flow chart summarizing the identification and selection of papers for systematic review.

Endnote software version X9 [13] (citation management software) was used to manage all research records exported from the databases. After making groups in the Endnote software by the name of the database (Embase, MEDLINE, or CINHAL), duplicates from both databases were removed from the Endnote file. This was followed by screening the unique studies obtained from both databases. Initially, all imported research articles in the Endnote software were screened by their respective titles. As a second step, the abstracts of the shortlisted articles were screened and read to assess whether any research study meets the eligibility criteria based on the abstract screening. In the end, the full text of those articles that were deemed eligible based on abstract screening was reviewed and read thoroughly. The entire process of selecting final eligible research studies was reported using the PRISMA flow diagram (Figure 1).

### 2.4. Process of Data Collection

A data extraction sheet was filed for eligible studies with full-text articles. The key parameters selected for the data extraction sheet included the study name/author, year of publication, country or setting where the study was conducted, study design, study population, sample size, diagnosis of the study participants, the age and gender of the study participants, prevalence or incidence of G6PD deficiency, risk factors or determinants of G6PD deficiency, key findings, and conclusions of authors.

## 3. Results

A total of 1099 records were identified in the databases. After removing 108 duplicates, 991 unique studies were left, whose titles and abstracts were screened. During this process of reviewing abstracts and titles, 605 abstracts and titles were found to be irrelevant and not related to the topic of interest at all. Hence, we had 386 eligible abstracts, of which 341 did not meet the full-text screening eligibility criteria, and full texts were not available for 22 citations. Consequently, 23 full texts were thoroughly read and reviewed for eligibility. Following a comprehensive review of the research articles based on the eligibility criteria, all 23 articles were incorporated into the review, as shown in Figure 1.

### 3.1. Study Characteristics

Studies were conducted in different Arab countries. More specifically, we had two studies each from Lebanon, Mauritania, and Bahrain (n = 2); one study each from Oman, UAE, and Sudan (n = 1); five from Egypt (n = 5); and three each from Iraq, Yemen, and Saudi Arabia (n = 3), as shown in Table 1. Regarding the year of publication, seven studies were published between 2000 and 2005, four studies between 2006 and 2010, eleven studies between 2011 and 2020, and one study was published in 2021. Overall, the sample size of the eligible research studies ranged from 31 to 48,889 diabetic patients for both males and females who participated in the respective studies. However, there was no equal proportion of males and females; rather, the proportion varied across the studies with varying proportions of G6PD deficiency. With respect to the type of study, there were mixed study designs. For example, there was one pilot study and case-series study each, three were community-based surveys, thirteen were cross-sectional studies, two were hospital-based studies, one was prospective, and two were retrospective studies. Almost all studies mentioned their outcome, which was mainly G6PD deficiency (Table 1). Also, it was found that almost all the included studies used validated and reliable methods to measure the outcome of interest.

### 3.2. Epidemiology of G6PD Deficiency in Arab Countries: Key Findings

Al-Arrayed et al. conducted a cross-sectional study on 11th-grade students from 38 schools in Bahrain. The study participants were 16–17 years old, 23.2% of the study participants were deficient for G6PD, and 1.9% were G6PD deficiency carriers. An almost equal proportion of males (11.4%) and females (11.9%) had G6PD deficiency. The authors found that the prevalence of G6PD deficiency was higher in geographic regions such as Sitara (45%), the western region (36%), Jidhafs (34%), the northern region (31%), and the central region (31%) [14]. There was a lower prevalence in areas such as Isa (17%), Muharraq (11%), Riffa (8%), and Hidd (5%). Among those who were G6PD deficient, 55% were females, and 45% were males [14]. Dash et al. conducted a hospital study in Bahrain in 2004. The study participants were 4173 newborns and 15,827 adults (n = 20,000) visiting the Salmaniya Medical Complex and various health centers of the Ministry of Health. The prevalence in Bahrain is the highest reported prevalence in the region, with twice the prevalence in males than females, and 1.4 times more in study subjects with sickle hemoglobin. More precisely, the authors found that G6PD deficiency was detected in 31.3% of the sample. Among males, the prevalence of the deficiency was higher at 40.6% than in females (23.1%), with a male-to-female ratio of 1.8:1. Male gender and sickle cell anemia were found to be the determinants of G6PD deficiency in this population [15].

Fattah et al. undertook a case-series study in Egypt on 69 neonates with pathological indirect hyperbilirubinemi. The mean age of the study participants was 36.78 ± 1.92 weeks, and 14.4% of the study participants were found to have G6PD deficiency. The male-to-female ratio was 2.33:1, and 21.2% of the males were affected, with a significantly higher proportion of males than females (*p*-value = 0.01). The authors also found that isolated G6PD deficiency was detected in 10% of neonates, mild deficiency was found in one patient, and three patients each had moderate and severe deficiencies [16]. Kasemy et al. conducted a cross-sectional study in Egypt on 487 neonates with jaundice and an average age of 4.45 ± 0.86 days. The authors found that G6PD deficiency was 10.1%, and males were 9.54 times more likely to develop G6PD deficiency than females. Male gender, family history, and consanguinity were found to be risk factors for G6PD deficiency. The authors found that mothers’ perceptions of G6PD deficiency were very low, and 17.10% had knowledge about G6PD deficiency. About 45% had a positive attitude toward G6PD deficiency, and 19.9% had good practice toward G6PD deficiency [17]. Elella et al. undertook a cross-sectional study in 2017 in Egypt on 2782 infants born in 2015. The authors found that 4.3% (119 infants) had G6PD deficiency, and 0.6% had an intermediate deficiency, with a male-to-female ratio of 3.2:1. About 6% (91) of males and 2.1% (28) of females had G6PD deficiency. Enzyme activity was significantly higher among males than females (*p*-value < 0.01) [18].

El Fotoh et al. conducted a prospective cross-sectional study on 202 neonates with indirect hyperbilirubinemia. Almost nine percent (8.9%) of the study participants had G6PD deficiency, and all males were G6PD deficient (100%). The authors also found that the mean serum bilirubin of G6PD deficient cases was 17.2 ± 4.4. A significant positive correlation was found between the time of appearance of jaundice in days and G6PD levels in deficient cases [19]. Hagag et al. conducted a study on 1000 patients with G6PD deficiency anemia. The authors found that males (932) were more commonly affected than females (68). A high prevalence of hemolytic crisis in G6PD deficiency was found among children 1–3 years old, with a mean age of 22.8 ± 15.54 months. Fava beans and falafel were the most common foods causing hemolysis, followed by chickpeas, broad beans, and green peas [20].

Al-Mendalawi et al. conducted a study in 2010 among 156 Iraqi children aged under five years who were G6PD deficient. The authors found that the ratio of males to females was 1.6:1 in Baghdad and 3.4:1 in Mosul. Family history was positive in 19.2% of the patients in Baghdad and 13.6% in Mosul [21]. Hilmi et al. undertook a study in 2002 on 758 (121 medical students and 69 hospital staff members (All males)) study participants aged 18–60 years. The prevalence of G6PD deficiency was 6.10%. The prominent non-deficient G6PD phenotype was G6PD B (92.6%), and 1.3% had G6PD A+. The presence of a substantial number of the non-Mediterranean variant was unexpected, and perhaps related to the more heterogeneous background of the Iraqi people. With respect to ethnicity, 6% of Arabs, 8.8% of Kurds, and 5.6% of Turkomans were G6PD-deficient [22].

Hassan et al. studied 1064 couples aged 14–60 years attending primary health care departments by conducting a cross-sectional study. About 12.5% of the study participants were deficient, and 15.3% of the males had G6PD deficiency. The prevalence of G6PD deficiency ranged from 11.4% in the Al-Madina district to 16.1% in the Abua al-Khasib district [23]. In 2006, Khneisser et al. undertook a cross-sectional study in Lebanon on 3000 men aged 14 years and above. The authors found that the cumulative incidence rate of G6PD deficiency was 12/1000, or 1.2%. The cases of G6PD deficiency were younger than the rest of the participants. Of those who were affected, 77.8% were aware of their problem since they were affected, and 22.2% were not aware of their problem [24]. In 2012, Innati et al. conducted a community-based survey in Lebanon on 3009 neonates. The prevalence of G6PD deficiency was 2.10%, with a higher prevalence among males (3.1%) than females (0.9%). Furthermore, there was a higher prevalence of G6PD deficiency among Muslims (2.6%) than among Christians (1.3%), as consanguinity is more common in Muslims [25]. In 2019, Djigo et al. conducted a cross-sectional study in Mauritania on 443 healthy blood donors. The prevalence of G6PD deficiency was 11.30%, and males had a higher prevalence (11.8%) than females (3.7%). Among males, Black Africans had the highest prevalence of G6PD deficiency (15%), and 5.9% of the White Moors were deficient [26].

In 2018, Mohamed et al. conducted a cross-sectional study in Mauritania on 523 neonates. The prevalence of G6PD deficiency was 11.09%, with a higher prevalence among males (15%) than females (7%). G6PD deficiency was more common in males than females (*p*-value = 0.007); also, Black children (15%) had a higher prevalence of G6PD deficiency than White children (8%) [27]. AL-Riyami et al. studied the prevalence of G6PD deficiency among 6103 Omani households and 6342 children aged 0–5 years. The prevalence of G6PD deficiency was 18.8%, with a higher prevalence among males (27%) than females (11%). There were no differences in the prevalence by age, but males had a higher prevalence than females. Also, prevalence varied according to the geographic region, with a higher prevalence in Al Dakhiliyah, followed by South Batinah, Muscat, and North Batinah [28]. In Saudi Arabia, Alharbi et al. conducted a study on 2100 Saudi men and found that 4.76% of the males were G6PD deficient. The G6PD A-mutation was present in 2% of the 100 subjects who were G6PD deficient. However, there was no significant difference in the frequency of this mutation between men with and without G6PD deficiency [29]. In 2005, Muzaffer et al. studied 2505 newborns in Saudi Arabia [30]. While the prevalence of G6PD deficiency was lower than in other studies (2%), the males appeared to have a higher burden of G6PD deficiency than females, with a male-to-female ratio of 3:1 [30]. Albagshi et al. conducted a retrospective study in Saudi Arabia in 2020 on 48,889 children aged 0–14 years that were admitted to a hospital. The prevalence of G6PD deficiency was 25%, 33.8% of the males were G6PD deficient, compared to 13.2% of females. The overall prevalence among all pediatric patients was 25%, whereas it was 18.8% in newborns, with a higher prevalence among males in both newborns and other kids [31]. In Sudan, Albsheer et al. conducted a cross-sectional study on 557 study participants. The authors found that 5.5% had severe or moderately severe G6PD deficiency. Factors such as body weight, region, and use of antibiotics were studied. However, in the multivariate model, body weight was found to be a significant protective factor, and a one-kilogram increase in body weight lowered the odds ratio of being G6PD deficient by 3% [32].

In 2000, Miller et al. undertook a cross-sectional community-based survey on 496 Emirati children aged 12–71 months. About 10% of the study participants were G6PD deficient, with one out of five females and four males [33]. The authors concluded that hereditary disorders—mainly G6PD deficiency—are common in Emirati children. G6PD deficiency was more common among male Emirati children than females [33]. Hussein et al. conducted a study in Yemen in 2011 on 90 adults aged 22 years. About 15% of the study participants were G6PD deficient, and among G6PD deficient participants, 75.5% were males and 24.5% were females. The authors further documented that 53.8% of the adults had a WHO class-II enzyme variant, 46.2% had a class-III variant, and no class-V variant was detected [34]. In contrast, 85.5% showed normal enzyme activity. Ghani et al. conducted a cross-sectional study in Yemen on 400 children residing in the malaria-endemic areas of the Hodeidah governate. The prevalence varied between 2.3–12.0% depending upon two varying cut-offs of ≤60 and ≤10%, respectively. The authors reported that 12.1% of children were G6PD deficient, and 2.3% were severely deficient [35]. Males had a higher prevalence of G6PD deficiency at all cut-off values, with significant differences at <20–40% of normal activity. Al Nood et al. conducted a pilot study in Yemen in 2009 on 31 patients with sickle cell trait aged 6mo–18 years. The prevalence of G6PD deficiency was 22.6%, with a higher burden of G6PD deficiency among patients with sickle cell anemia [36] (Table 2).

## 4. Discussion

The current systematic review was undertaken to provide insights into the epidemiology of G6PD deficiency in Arab countries. The study findings from the review revealed that different countries experience a varying burden of G6PD deficiency. Overall, the prevalence of G6PD deficiency ranged from 2 to 31%, with a higher burden of G6PD deficiency found among high-risk populations such as neonates with sickle cell anemia. The determinants of the G6PD deficiency studied in these studies included males, family history, consanguineous marriages, geographic regions, and body weight. All of the mentioned factors were risk factors for G6PD deficiency except body weight, which was a protective factor. The universal finding across all studies was a higher prevalence of G6PD deficiency among males than females, with a ratio of at least 3:1.

Glucose-6-phosphate dehydrogenase enzyme contributes to the pentose phosphate pathway, which generates NADPH, which then maintains the reduced glutathione [37,38]. The reduced glutathione is a protective mechanism against oxidative damage in red blood cells [37,38]. The scarcity of G6PD increases the fragility of red blood cells, resulting in hemolysis [37,38]. Clinical, biochemical, and molecular heterogeneity characterizes G6PD deficiency with varying prevalence across the globe [7,37,38]. In general, G6PD deficiency is the most common enzyme deficiency around the world, with a substantial variation in the prevalence across regions. Overall, the literature suggests that Arab countries have the second highest prevalence of G6PD deficiency after African countries [7].

Since G6PD deficiency is an X-linked recessive disorder, the finding regarding males being more commonly affected than females is not surprising, and this finding is universal and consistent with almost all studies conducted across the world [38,39]. The males are found to be either hemizygous wild types, or have a mutation for the G6PD gene. The mutant G6PD gene results in G6PD deficiency [39]. On the other hand, females are less likely to develop G6PD deficiency because, for females, there are two copies of the G6PD gene, and mutation in both copies (homozygous) can lead to G6PD deficiency [37,39]. Those females with one wild type and one mutant allele are considered heterozygous for the G6PD gene [39]. Furthermore, the expression of gene mutation is more common in males because favorable X-chromosome inactivation causes heterozygous females to likely not be deficient [39,40].

Similarly, the prevalence of G6PD deficiency was high among couples who had consanguineous marriages. Commonly, some people tend to marry their first or second cousins (consanguineous unions; i.e., family endogamy), and family endogamy is common in Arab countries [41,42]. Since family endogamy has been considered a causal factor in the prevalence of genetic disorders, it increases the chances of homozygosity, and therefore increases the probability of G6PD deficiency [43].

While the guidelines for screening and diagnostic testing may differ by geographical location, the World Health Organization recommends that countries with a prevalence of 3–5% among males should screen newborns routinely [44]. Hence, Arab countries with a higher G6PD deficiency among males may need to introduce a national newborn screening program for the timely detection of the condition and proper management of the infants. For example, if the disease is detected on time, the newborns can undergo phototherapy, a simple and cost-effective strategy, to prevent newborns from developing kernicterus. Also, after detecting the disease on time, major health problems or hazards of transfusing blood can be eliminated.

### Strengths and Limitations

This is a unique review, as it is the first of its type that provides insights into the epidemiology of G6PD deficiency in Arab countries. We used an updated PRISMA checklist to conduct this review, and a quality assessment of the eligible studies was performed using appropriate scales, as per the study design. Moreover, we provided insights from 23 studies, which reflects enough of a sample size of the studies; however, not all Arab countries had equal representation due to the lack of studies in a few countries. The findings of this review can provide a framework for clinicians, pediatricians, neonatologists, and policymakers to plan for routine newborn screening to detect G6PD deficiency in a timely manner.

Despite these strengths, the findings of the review should be interpreted with caution due to the limitations of the eligible studies included in the review. First, due to the non-random selection of study participants in the selected studies of the review, study findings may only be generalized to some of the population. Second, an issue of unmeasured confounding cannot be ignored due to the observational nature of the study designs included in the review. In addition, not all studies used universal cut-offs to detect enzyme deficiency or the inactivity of an enzyme. Though authors used standard methods to detect G6PD deficiency, there was a lack of consistency in using methods. While the majority of the studies were cross-sectional or community-based surveys, there were few studies conducted on hospitalized populations or high-risk groups that skewed the prevalence to the higher side.

Given the limitations of the existing review, more robust evidence is required, with a relatively larger number of studies warranted in the future from all Arab countries with a random selection of study participants. However, the findings of the current review may help researchers and clinicians learn about the burden of G6PD deficiency and its associated risk factors and gender distribution in different Arab countries.

## 5. Conclusions

The prevalence of G6PD deficiency varies across Arab countries, with a higher prevalence in males than females. Males, children with a family history, or children of couples with consanguineous marriages were at a higher risk of developing G6PD deficiency than their counterparts. Different regions of Arab countries need to revisit the screening and diagnostic guidelines to detect G6PD deficiency in a timely manner and prevent unnecessary morbidity and mortality among their communities.

## Figures and Tables

**Table 1:** Characteristics of the studies included in the systematic review (n = 23).

Author	Year	Country	Study Design	Sample Size	Target Population	Age
Al-Arrayed et al.	2003	Bahrain	Cross-sectional	5686	11th-grade students from 38 schools	16–17 years
Dash et al.	2004	Bahrain	Hospital-based study	4173 newborns, 15,827 adults	Bahrainis visiting the Salmaniya Medical Complex and various health centers of the Ministry of Health.	Not specified
Fattah et al.	2010	Egypt	Case-series study	69	Neonates with pathological indirect hyperbilirubinemia	36.78 ± 1.92 weeks
Kasemy et al.	2019	Egypt	Cross-sectional	487	Neonates with Jaundice	4.45 ± 0.86 days
Elella et al.	2017	Egypt	Cross-sectional	2782	Infants born during 2015	Under one year
El Fotoh et al.	2016	Egypt	Prospective cross-sectional	202	Neonates with indirect hyperbilirubinemia	3.7 ± 2.5 days
Hagag et al.	2017	Egypt	Retrospective study	1000	Patients with G6PD deficiency anemia	22.8 ± 15.54
Al-Mendalawi et al.	2010	Iraq	Cross-sectional	156	Children under 5 years	2.8 ± 1.2 years
Hilmi et al.	2002	Iraq	Cross-sectional	758	121 medical students and 69 hospital staff members (All males)	18–60 years
Hassan et al.	2003	Iraq	Cross-sectional	1064	Couples aged 14–60 years attending a primary health care department	14–60 years
Khneisser et al.	2006	Lebanon	Community-based survey	3000	Men	14 years and above
Inati et al.	2012	Lebanon	Cross-sectional	3009	neonates	<1 month
Djigo et al.	2019	Mauritania	Cross-sectional	443	Healthy blood donors	Median age: 30 years (18–61)
Mohamed et al.	2018	Mauritania	Cross-sectional	523	Newborns	<1 month
AL-Riyami et al.	2001	Oman	Community-based study	6342	The general population (children)	0–5 years
Alharbi et al.	2014	Saudi Arabia	Cross-sectional	2100	Saudi Men	43 ± 10.05
Muzaffer et al.	2005	Saudi Arabia	Hospital-based study	2505	Newborns	<1 month
Albagshi et al.	2020	Saudi Arabia	Retrospective study	48,889	Children 0–14 years	1.93 ± 3.98
Albsheer et al.	2021	Sudan	Cross-sectional	557	The general population from New Halfa and Khartum	34.2 ±15.6
Miller et al.	2000	UAE	Cross-sectional community-based survey	496	Emirati children	12–71 months
Hussein et al.	2011	Yemen	Cross-sectional	90	Adult individuals	22 years
Ghani et al.	2016	Yemen	Cross-sectional	400	Children residing in the malaria endemic areas of the Hodeidah governate	Eight years (6–11 years)
Al Nood	2009	Yemen	Pilot study	31	Patients with sickle cell trait	6 months–18 years

**Table 2:** Key findings on the epidemiology of G6PD deficiency in Arab countries (n = 23).

Author	Prevalence	Gender-Specific Prevalence	Risk Factors	Key Findings
Al-Arrayed et al.	23.2% for G6PD deficiency and 1.9% G6PD deficiency carriers	Males: 11.4%	Not studied	Prevalence was higher among geographic regions such as Sitara (45%), the western region (36%), Jidhafs (34%), the northern region (31%), and the central region (31%). Prevalence was lower in areas such as Isa (17%), Muharraq (11%), Riffa (8%), and Hidd (5%). Among those who were G6PD deficient, 55% were females, compared to 45% were males.
		Females: 11.9%		
Dash et al.	G6PD deficiency was detected in 31.3% of the sample	G6PD deficiency among males: 40.6%	Gender and sickle cell anemia	The prevalence in Bahrain is the highest reported prevalence in the region, with twice the prevalence in males than females and 1.4 times more in study subjects with sickle hemoglobin.
		Females: 23.1%		
		G6PD deficiency M: F ratio: 1.8:1		
Fattah et al.	14.40%	21.2% in males, with a significantly higher proportion in males than females (0.01). Male to female ratio was 2.33:1.	Gender	Isolated G6PD deficiency was detected in 10% of neonates, mild deficiency was found in one patient, and three patients each had moderate and severe deficiency.
Kasemy et al.	10.10%	Males were 9.54 times more likely to develop deficiency than females.	Male gender, family history, and consanguinity	Mothers’ perceptions of G6PD deficiency were very low, and 17.10% had knowledge about G6PD deficiency. About 45% had a positive attitude toward G6PD deficiency, and 19.9% had good practice toward G6PD deficiency.
Elella et al.	4.3% (119 infants) had G6PD deficiency, and 0.6% had an intermediate deficiency	Male to female ratio of 3.2:1.	Not studied	About 6% (91) of males and 2.1% (28) of females had G6PD deficiency. Enzyme activity was significantly higher among males than females (*p* < 0.01)
El Fotoh et al.	8.9% had G6PD deficiency	All males were G6PD deficient (100%)	Gender	The mean serum bilirubin of G6PD deficient cases was 17.2 ±4.4. A significant positive correlation was found between the time of appearance of jaundice in days and G6PD levels in deficient cases.
Hagag et al.	93.2% in males and 6.8% in females.	Males (932) were more commonly affected than females (68)	Gender. Diet and infections were the common causes of hemolysis	A high prevalence of hemolytic crisis in g6PD deficiency was found among children 1–3 years old with a mean age of 22.8 ± 15.54 months. Fava beans and falafel were the most common foods causing hemolysis, followed by chickpeas, broad beans, and green peas
Al-Mendalawi et al.	Not applicable, as all patients included had G6PD deficiency	The ratio of males to females was 1.6:1 in Baghdad and 3.4:1 in Mosul	Family history and male gender	Family history was positive in 19.2% of the patients in Baghdad and 13.6% in Mosul.
Hilmi et al.	6.10%	Not applicable, as all were males	Not studied	The prominent non-deficient G6PD phenotype was G6PD B (92.6%), and 1.3% had G6PD A+. The presence of a substantial number of the non-Mediterranean variant was unexpected and perhaps related to the more heterogenous background of the Iraqi people. With respect to ethnicity, 6% of Arabs, 8.8% of Kurds, and 5.6% of Turkomans were G6PD-deficient.
Hassan et al.	12.50%	Males: 15.3%	Not studied	The prevalence of G6PD deficiency ranged from 11.4% in Al-Madina to 16.1% in Abua al-Khasib.
Khneisser et al.	Cumulative incidence rate: 12/1000 or 1.2%	Not applicable, as all were males	Not studied	Cases of G6PD deficiency were younger than the rest of the participants.
				Of those who were affected, 77.8% were aware of their problem since they were affected, and 22.2% were not aware of their problem.
Inati et al.	2.10%	Males: 3.1%	Male gender and Muslims	There was a higher prevalence of G6PD deficiency among Muslims (2.6%) than among Christians (1.3%), as consanguinity is more common in Muslims.
		Females: 0.9%		
Djigo et al.	11.30%	Males: 11.8%	Not studied	Among males, Black Africans had the highest prevalence of G6PD deficiency (15%), and 5.9% of White Moors were deficient.
		Females: 3.7%		
Mohamed et al.	11.09%	Males: 15%	Black children and males	G6PD deficiency was more common in males than females (*p* = 0.007); also, Black children (15%) had a higher prevalence of G6PD deficiency than White children (8%).
		Females: 7%		
AL-Riyami et al.	18.80%	Males: 27%	Gender and geographic region	There were no differences in the prevalence by age, but males had a higher prevalence than females. Also, prevalence varied according to the geographic region, with a higher prevalence in Al Dakhiliyah, followed by South Batinah, Muscat, and North Batinah.
		Females: 11%		
Alharbi et al.	4.76%	Not applicable, as all were males	Not studied	In total, 100 males (4.76%) were found to have G6PD deficiency. The G6PD A-mutation was present in 2% of the 100 subjects who were G6PD deficient. However, there was no significant difference in the frequency of this mutation between men with and without G6PD deficiency.
Muzaffer et al.	2%	Males: 3.05%	Male sex	Six babies (5 males and 1 female) developed neonatal jaundice
		Females: 0.9%		
		Male to female ratio: 3:1		
Albagshi et al.	25%	Males: 33.8%	Male sex	The overall prevalence among all pediatric patients was 25%, whereas it was 18.8% in newborns, with a higher prevalence among males in both newborns and other kids.
		Females: 13.2%		
Albsheer et al.	Low-to-moderate but with high heterogeneity. 5.5–27.3%	Males: Median G6PD activity is 4.2 compared to 4.3 in females.	Body weight, region, and use of antibiotics were studied, and body weight was found to be a significant protective factor	5.5% had severe or moderately severe G6PD deficiency. One kg increase in body weight lowered the OR of being G6PD deficient by 3%.
Miller et al.	9.10%	One out of five were females, and one out of four were males	Not studied	Hereditary disorders, mainly G6PD deficiency, are common in Emirati children. G6PD deficiency was more common among male Emirati children than females.
Hussein et al.	14.50%	Among those who were deficient, 75.5% were males, and 24.5% females	Not studied	53.8% of the adults had a WHO class-II enzyme variant, 46.2% had a class-III variant, and no class-V variant was detected. Whereas 85.5% showed normal enzyme activity
Ghani et al.	Prevalence varied between 2.3–12.0% depending upon two varying cut-offs of ≤60 and ≤10%, respectively	Males: 2.7–14.2%	Male gender, district of residence and consanguineous marriage	12.1% of children were G6PD deficient, and 2.3% were severely deficient.
		Females: 1.7–9.4%		Males had a higher prevalence of G6PD deficiency at all cut-off values with significant differences at <20–40% of normal activity
Al Nood	22.60%	Not reported	Not studied	The burden of G6PD deficiency was high among patients with sickle cell anemia.

## Data Availability

All data analyzed during this systematic review are included. Additional data are available from the corresponding author upon reasonable request.
